# An efficient record linkage scheme using graphical analysis for identifier error detection

**DOI:** 10.1186/1472-6947-11-7

**Published:** 2011-02-01

**Authors:** John M Finney, A Sarah Walker, Tim EA Peto, David H Wyllie

**Affiliations:** 1 NIHR Biomedical Research Centre, John Radcliffe Hospital, Oxford, UK; 2 MRC Clinical Trials Unit, London, UK

## Abstract

**Background:**

Integration of information on individuals (record linkage) is a key problem in healthcare delivery, epidemiology, and "business intelligence" applications. It is now common to be required to link very large numbers of records, often containing various combinations of theoretically unique identifiers, such as NHS numbers, which are both incomplete and error-prone.

**Methods:**

We describe a two-step record linkage algorithm in which identifiers with high cardinality are identified or generated, and used to perform an initial exact match based linkage. Subsequently, the resulting clusters are studied and, if appropriate, partitioned using a graph based algorithm detecting erroneous identifiers.

**Results:**

The system was used to cluster over 250 million health records from five data sources within a large UK hospital group. Linkage, which was completed in about 30 minutes, yielded 3.6 million clusters of which about 99.8% contain, with high likelihood, records from one patient. Although computationally efficient, the algorithm's requirement for exact matching of at least one identifier of each record to another for cluster formation may be a limitation in some databases containing records of low identifier quality.

**Conclusions:**

The technique described offers a simple, fast and highly efficient two-step method for large scale initial linkage for records commonly found in the UK's National Health Service.

## Background

Integration of information on individuals (record linkage) is a key problem in healthcare delivery, epidemiology, and "business intelligence" applications[1-3]. Large data sources are increasingly available in many areas, but unfortunately accurate and ubiquitously applied unique identifiers are rarely available. Frequently, identifiers which are supposed to be unique to an individual (e.g. UK National Health Service numbers, or the patient numbers generated by hospitals) are missing from some or all data items; additionally, some are incorrectly entered due to clerical or typographic errors.

Identifier inaccuracies can result in both organisation costs and risks to individuals.

Because of the problem of identifier error, in many processes (such as the identification of samples sent to hospital laboratories) the record to be linked is usually identified by multiple pieces of information, such as a name and date of birth as well as the supposedly unique identifying number.

Approaches mapping records with multiple identifiers to individuals have been extensively studied[4]. In a classical, probabilistic approach, after similarities between identifiers (such as surnames, forenames, and so on) have been computed using functions such as the Levenhstein distance[5] and Jaro-Winkler functions [4], Bayesian classification is used to discern likely matches[6]. In general, probabilistic approaches can require up to (*n*(*n*-1)/2) comparisons to merge two files each containing *n *records, and so prove unfeasibly expensive computationally for the very large, dynamic datasets available in many situations. This complexity requires use of heuristics to divide the database into smaller sections (termed "blocks", or "canopies") within which comparisons can be made. Since linkage only occurs within each small section, the algorithm dividing the database can prevent linkage if it does not assign all an individual's records to the same block[4,7]. Nevertheless, it has been shown that carefully optimised heuristics combining exact and probabilistic matching can be used to generate large databases of healthcare records with good performance[8], especially when good quality unique identifiers (e.g. NHS number) can be used in initial linkage steps.

There is growing interest in the use of graphs, data structures in which items of data are represented as nodes and their similarities as edges, to store complex relationships in general[9]. An important example is the work of Sauleau and colleagues [10], who considered the problem of 300,000 clustering health records from a French hospital. They used an approach derived from probabilistic clustering literature on canopies (overlapping blocks) of records, generating pairwise distances between each record. In contrast to classical probabilistic linkage, they then considered records as nodes, and their pairwise distances as weighted edges in an undirected graph from which records of similar patients can be recovered using a hard clustering method (i.e. cluster membership is binary, not probabilistic). In their approach, assignation of edge weight is a critical step. Another sophisticated example is the work of Kalashnikov and colleagues [11]. They investigated clustering based on paths of connectivity between identifiers which are not themselves unique, but the strength of whose connections can be determined by path analysis, and where the optimal cluster edges can be determined by minimizing edge weights.

In many important 'real-world' situations, including healthcare, identifier(s) are available for each record which would be expected to be unique to an individual. These would include 'purpose built' identifiers, such as hospital numbers, but also identifiers comprising high-cardinality combinations of personal data (surname, forename, home telephone number), or (surname, forename, date of birth). Additionally, in 'real world' situations, these identifiers contain errors at some low frequency. In commonly used systems for allocating identifying numbers, particularly sequential allocation, typographical errors in one identifier number may not only result in records containing a novel identifier, but also may generate another person's identifier with high probability. As numbers of records increase, these errors become increasingly important, producing theoretically unique identifiers in records genuinely belonging to two or more different individuals. The existence of this type of error detracts from otherwise appealing and efficient exact-match record linkage methods combining records sharing unique identifiers, as the identifier errors cause co-clustering of records from different individuals, referred to here as record collision, compromising overall linkage quality.

Here we describe a simple and highly efficient solution to the identifier-collision problem, in which collisions are detected by noting discrepancies in unique identifiers within collision-affected records. Our research was driven by the need to link accurately more than 250 million health care records from large UK hospitals for clinical and epidemiological purposes.

## Methods

### Task definition

From large sets of health records (such as patient admissions and laboratory samples), each of which is identified by one or more pieces of personal data, the objective is to assign records to the individuals from whom they originated in an efficient, sensitive and specific manner. In this setting, we considered that a 'masterlist' or 'gold standard' data set of all patients is not available, requiring that the individuals be discerned from the identifiers present in the record set. Computational efficiency is important, since rapid performance is desirable for clinical decision making. So too is sensitivity and specificity, where sensitive linkage refers to assigning the records to the correct individual; specificity refers to assigning all records from a single individual to a single individual.

### Overview of algorithm

We present an algorithm which has the following components, which are described in subsequent sections:

• Identifier cleaning (Section 1)

• Construction of high-cardinality identifiers from combinations of identifiers, such as forename, surname and date of birth (Section 2)

• Exact match using the constructed high-cardinality identifiers (Section 3)

• Detection of clusters containing more than one individuals 'identity collision' using logistic classification, applied to all clusters containing any variation in identifiers; (Section 4)

• Breaking apart of clusters with identity collision (Section 5)

### Data set available

We had available medical records from each of five data sources: the patient administration system (PAS), details of any previous names patients may have had (PAShistory), an emergency admission tracking system (Jonah), a microbiology information system (Micro) and a haematology, biochemistry and immunology laboratory information management systems (LIMS) of a large UK hospital, covering about 1% of the population of the United Kingdom.

The laboratory systems (LIMS and Micro) provide services to the hospital, but also to a large number of general practitioners supplying outpatient samples, samples which make up about half the workload. The outpatient workload is not necessarily represented in the PAS system; therefore, a 'masterlist' of patients does not exist prior to linkage.

Personal information available included forename, surname, gender, date of birth, NHS number, and an identification number generated by the hospital, although, as discussed below, not all records contained complete information. Data sources used started on 1/1/1994 (LIMS), 1/1/1997 (Micro), 1/4/1997 (PAS & PasHistory), 1/1/2005 (Jonah) and ended on 30 March 2010. An example of the data available is shown in Table 1. In total, these data sources contain over 250 million records.

**Table 1:** Identifiers and Record linkage operation

Start cluster id	New cluster id	NHS number	hospital number	Surname	Forename	sex	date of birth (ddmmyyyy)	frequency of occurrence
1	1	NULL	4496644	WILSON	DAVID	M	14061940	3
2	2	5170231111	NULL	WILSON	DAVID	M	01051939	1
3	3	3319004037	4118890	WILSON	DAVID	M	20011969	2
4	4	NULL	NULL	WILSON	DAVID	M	20011969	1
5	3	3319004037	NULL	WILSON	DAVID	M	20011969	2
6	6	NULL	4118890	WILSON	DAVID	M	20011969	1

An example of identifiers provided for patients with forename and surname 'David Wilson'. The details have been changed to protect patient confidentiality. Null fields indicate there was no information provided in that field.

One cycle of the record linkage is illustrated. Consider each combination of identifiers to belong to its own, discrete cluster, identified by a cluster identifier (Start cluster id). For all sets in which at least one member shares an NHS number identifier with a different set, combine these sets into a single set (New Cluster ID). The operation proceeds for all identifiers.

### Data platform used and Statistical software

We stored the data on a single Windows Server 2003 running SQL Server 2005 databases, with Dell hardware, two 2.5 GHz Xeon processors (8 cores total), 1.2TB RAID 5 hard disc space, and 16GB of RAM. Linkage algorithms were implemented as SQL server stored procedures. Jaro-Winkler and Levensthein distance calculations were implemented using the Simmetrics package[12], compiled into the SQL server using CLR integration.

### Clustering operations

Here, we describe the stages of the clustering operation used.

### 1 Data cleaning

We set to null identifiers which did not pass a series of quality control checks, which were implemented with custom C# functions. We also removed (eliminated from the string identifier) certain patterns, such as the forename "BABY". The checks were based on domain knowledge within the group, and are summarised in Table 2. Regarding hospital numbers, if different hospitals could issue the same hospital number to different individuals, the hospital number is prefixed by a code which makes it unique to that hospital, e.g. number 123456 from hospital A is adjusted to 'A:123456' or similar.

**Table 2:** Identifier cleaning

All fields	Fields converted to uppercase blanks (e.g. whitespace) deleted	All fields
Forename & Surname	remove of forenames containing baby/infant/twins, or synonyms. Remove all symbols, e.g. '. deletion of records matching internal hospital test individuals. removal of non-alphabetic values remove values containing only one letter reverse forename and surname if stated forename does not exist in any patient administration records as a forename	Forename & Surname
Sex	Remove unless M, F, U characters, representing male, female or unknown, respectively	Sex
Hospital numbers	Remove checkdigits Remove out-of-range numeric values Deleted, along with all other identifiers, if the patient is from a Genito Urinary medicine clinic, or from the Occupational Health Department.	Hospital numbers
NHS numbers	Delete out-of-range values Delete values not conforming to checkdigit requirement as described: http://www.datadictionary.nhs.uk/data_dictionary/attributes/n/nhs_number_de.asp	NHS numbers

Birthdate & Deathdate	Conversion to SQL date format remove dates before 1860-01-01 remove dates in the future	Birthdate & Deathdate

The steps taken in cleaning data items are described.

### 2 Construction of high cardinality identifiers by concatenation

We considered whether concatenation of existing identifiers, particularly surname, forename and date of birth, might offer an identifier of high cardinality with potential to act as a unique identifier, using records in the PAS data source having NHS numbers. This subset represents recent PAS entries and is more heavily curated than other data sources, due to cross-checking against central NHS identity databases via the NHS's Spine infrastructure. It represents the subset of patients with contact with the hospital, which we do not believe to differ systematically in terms of names and dates of birth from those without. We counted distinct NHS numbers (chosen because it is supposed to be unique to an individual) mapping to particular combinations of name and date of birth.

### 3 Initial Record linkage using identifiers

a) We used an initial linkage algorithm which joins all records having any of three high-cardinality identifiers in common, using an iterative procedure one set of which is described in Table 1. The operation ceases when no set of records contains a shared identifier. The sets of records thus formed are termed clusters.

Considering complexity, the implementation is possible using SQL; for one identifier (such as NHS number), in a table with n rows, identifying the records sharing a common identifier can be implemented as a hash join of the table to itself on the identifier, and has complexity O(n), where n is the number of rows to be analysed; the operation has to be repeated across all m identifiers (3, in this case), so complexity for one operation (b) is O(mn) [13,14]. Because of the nature of set union operations, the order of these operations do not matter, and the solution found is deterministic. The number of iterations required to complete linkage depends on the combinations of identifiers present within the clusters. If the number of shared identifiers remaining after the first set union is very small relative to the total number of records, then overall complexity is approximated by O(mn) as the number of iterations is 1 for almost all records.

### 4 Identity collision and its detection

It is possible for records from more than one individual to be combined into the same cluster; this results in intra-cluster variation, and is discussed in Results. First, we studied the variation occurring within the clusters produced by initial linkage. We did this by identifying a random sample of 25,000 "complex" clusters was obtained, defining "complex" to mean any difference in any of the identifiers in section 2 within the records comprising the cluster, i.e. having intra-cluster variation.

Some of the intra-cluster variation arises from variations which occur in identifiers, e.g. on marriage. Other variation arises from combination of individuals. We devised a sensitive test for clusters belonging to one person, based on domain knowledge: we provisionally defined regarded the 25,000 clusters as 'good' if they had

• one NHS number and one hospital number, or

• one NHS number, one name and date of birth

• one hospital number and one name and date of birth

We then simulated clusters resulting from inappropriate combination (termed 'bad' clusters) of two 'good' clusters (defined operationally as above) by randomly combining pairs of 'good' clusters. The artificially formed bad clusters generated in this way resemble one frequently observed pattern of mislinkage, as is described in Results.

Thus, three groups of records were derived: 'good', meeting the above criteria, 'uncertain', which were present in the original dataset but did not meet the above criteria, and 'bad' which are derived from good clusters by simulation.

We considered 'good' and 'bad' clusters further. We computed the maximum distance between the all combinations of the fields in the cluster and fitted logistic regression models for clusters including females (excluding distances based on surname, because of the frequent changes in surname occurring on marriage), or for clusters without any females, modelling the odds of bad status relative to good. This process used R scripts calling SQL server stored procedures to extract and simulate clusters, followed by backwards conditional logistic regression modelling with the stepAIC function from the R MASS package[15]. The distribution of scores was plotted using the R lattice[16] densityplot function for 'good' and 'bad' clusters, as well as for clusters considered uncertain by rule-based classification. Cutoffs were chosen by visual inspection of density plots and performance of the fitted model assessed on an independent simulation, extracted as above.

### 5 Identity collision resolution

We hypothesised that the major cause of 'bad' clusters - the co-clustering of records derived from two different individuals - was the presence of a mistake, perhaps due to typographical error - in a *single *identifier. If this is true, then

**Figure 1:**
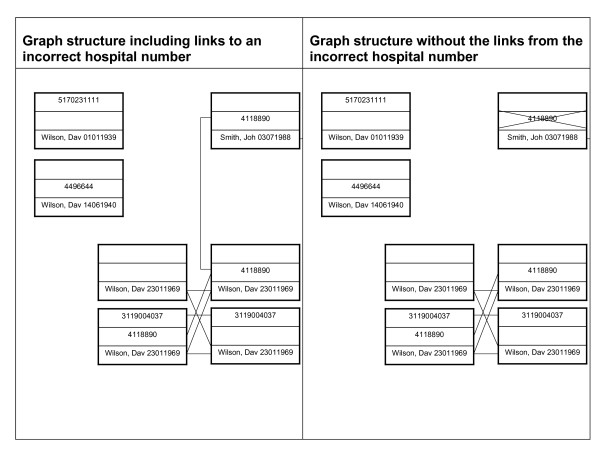
**Collision of two clusters**. The collision described in Table 6 is illustrated graphically. A single identifier joins two clusters containing records from two patients. When edges are not formed from this (right panel) the clusters are no longer joined.

1. identifiers whose removal leads to the division of a cluster into two, where the two divided clusters have improved 'quality' relative to the initial cluster are potentially bad. An example is illustrated in Figure 1.

2. When one views the identifier combinations as a graph, with edges comprising shared identifiers (Figure 1), the erroneous identifiers form the origins of edges.

3. It follows that potentially erroneous identifiers are only a small subset of all the identifiers in the records of interest, in that they both form the origins of edges (as in 2, above); further, that they lie within the set of identifiers having the properties in (1, above).

### 6 Trial of a splitting heuristic by simulation

An unresolved issue is how 'increased quality', referred to above, is to be measured. Various measures are possible. Given that the cluster is highly likely to be bad before identity collision resolution, we experimented with a simple heuristic measure of quality. We considered our three identifiers used for clustering, and their within-cluster cardinality (that is, the number of variants within them). We hypothesised that maximizing the increase in invariant identifiers (i.e. those with cardinality 1) on identifier deletion might allow splitting of clusters into their two constituent parts. This was tested by simulation on one of the identifiers, hospital number.

a) Pairs of randomly selected 'good' clusters were combined, replacing one randomly selected hospital number from one cluster with the maximal hospital number from the other, thus simulating a cluster collision caused by entry of a 'bad' hospital number.

We then tested a cluster splitting algorithm, as follows:

b) variants of the cluster were generated, and in each variant one identifier was deleted; the variant was then re-clustered (described above in Initial record linkage) performed.

c) We identified variants generated in b) in which maximised the increase in identifiers with cardinality = 1. If more than one variant had this property, we identified one at random.

d) We scored the cluster splitting as successful if the identifier picked for deletion on the basis that it was bad in step (c) was the same 'bad' identifier selected in step (a).

### 7 Fuzzy Search

We used two fuzzy search algorithms, together with manual curation, to estimate the proportion of clusters which are highly similar. Firstly, to find records similar to a query record, we determined all trigrams (substrings containing three consecutive characters) of surname, forename, date of birth, hospital and NHS numbers within the query[17], and identified the top 10 matches by ranking matches according to the numbers of shared trigrams between the query record and all other records in the database. Secondly, we identified the subset of records having identical first surname and forename to the query record. After both approaches, the candidates generated were scored as 'likely to be from the same patient' or 'not likely to be from the same patient' subjectively by two different observers.

## Results

### Identifiers with high cardinality

We wished to link a large number of health records both efficiently and specifically; examples of the identifiers available are shown in Table 1. The data set included hospital and NHS numbers, which are intended to uniquely identify patients; however, other fields do not. We found than an identifier could be constructed from combinations of name, surname and date of birth with cardinality comparable with NHS number. Name and surname alone has somewhat less cardinality (Table 3).

**Table 3:** High cardinality of combinations of name and date of birth

Identifier	Cardinality	Average NHS numbers per identifier
National Health Service Number	1066339	1
Date of birth	35694	29.87
Surname, complete forename	829650	1.285
Surname, first letter of forename, date of birth	1065027	1.001316
Surname, first three letters of forename, date of birth	1066184	1.000234
Surname, complete forename, date of birth	1066519	1.000090

For a set of complete identifiers from one data source (PAS), we show the cardinality (number of discrete values) for each of a series of possible identifiers, and average number of NHS numbers per identifier. This cardinality of NHS number and combinations of name and date of birth are similar.

### Incomplete identifiers

We therefore investigated NHS number, hospital number, and the combination of the first three characters of the forename, whole surname and date of birth further as a potentially unique identifier (Table 3). Following data cleaning (see Methods), at least one valid identifier was available for almost all samples from data sources other than the LIMS system, where historical data was of had low identifier frequency due to laboratory protocols then in place, and microbiology, where 12.8% have no identifiers. These latter samples represent samples from Genito-urinary medicine departments, and deliberately lack identifiers, as is required by statute in the United Kingdom (Table 4). Records without any valid identifiers were not considered further.

**Table 4:** Combinations of identifiers available on different record sources

Hospital Number	NHS Number	Name/date of birth	jonah	lims	micro	pas	pashistory	Total
+	+	+	456553 (72.2%)	246326 (3.6%)	1494645 (28.5%)	1205042 (53.3%)	94935 (83.4%)	1494645 (16.2%)
+	+	-	223 (0%)	1510 (0%)	5520 (0.1%)	19086 (0.8%)	161 (0.1%)	19086 (0.2%)
+	-	+	174874 (27.7%)	2160475 (31.7%)	978448 (18.6%)	860916 (38.1%)	18372 (16.1%)	2160475 (23.4%)
+	-	-	550 (0.1%)	30816 (0.5%)	36636 (0.7%)	177518 (7.8%)	366 (0.3%)	177518 (1.9%)
-	+	+	7 (0%)	103420 (1.5%)	813906 (15.5%)	0 (0%)	0 (0%)	813906 (8.8%)
-	+	-	0 (0%)	591 (0%)	2244 (0%)	0 (0%)	0 (0%)	2244 (0%)
-	-	+	95 (0%)	3883941 (57%)	1245979 (23.7%)	1 (0%)	1 (0%)	3883941 (42.1%)
-	-	-	3 (0%)	382490 (5.6%)	671076 (12.8%)	0 (0%)	0 (0%)	671076 (7.3%)

Up to three identifiers, hospital number, NHS number and name & date of birth are available for each record, but they are not all present in each data set. Shown are the combinations of identifiers (- = absent,+ = present) for each dataset contributing to the database.

### Initial Record linkage using identifiers

**Figure 2:**
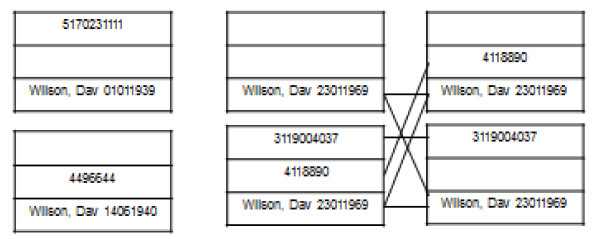
**Representation of data as a graph**. Above is shown the result of one real cluster generated by the algorithm; to protect patient confidentiality, patient details have been replaced by example details. Here, there are three discrete patients, all called David Wilson, but differing in dates of birth, NHS and hospital numbers. Edges join nodes having shared identifiers.

We considered combinations of identifiers (e.g. each row in Table 1) as the unit to be linked. We used an algorithm which joins all records having any common identifier, implemented using set-based operations (see Methods), although we note that the operation can also be viewed as a graph clustering operation. In particular, one can view identifier combinations as vertices V in an undirected incomplete graph G, with an edge between vertices present if they share an identifier, and cliques as clusters containing results from an individual (Figure 2). After initial linkage, we found 3,557,951 clusters. Of these, 284,636 (8.2%) had more than one identifier in any of NHS number, hospital number, and the combination of the first three digits of the forename, surname and date of birth.

### Identity collision and its detection

Despite the efficiency of the initial clustering operation, it has a major problem. If an identifier is mistyped, and happens to be an identifier used by another individual, then these two individual will be assigned to the same cluster. This process ("collision") can generate differences in multiple identifiers (e.g. in surname, forename, date of birth) between elements in the set generated by identity collisions. We used these to these distances to build logistic regression models predicting that the clusters created by initial exact match clustering contained multiple individuals.

**Figure 3:**
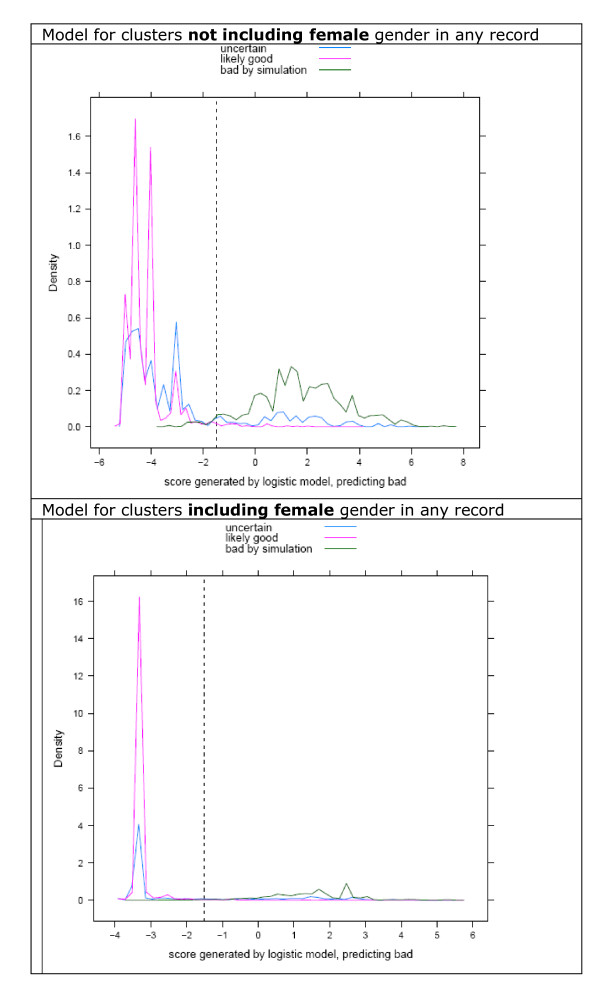
**Classification of data into good and bad clusters**. A random sample of 25,000 complex clusters was obtained after initial record linkage. Complex clusters are those with more than one variant of at least one identifier. These clusters were divided into those which, on the basis of a series of rules, were thought to represent one individual ('likely good', purple line), or the others (uncertain, blue line). Good clusters were then combined randomly creating a new set of clusters (bad by simulation, green line). Maximal distances were computed for pairwise distances within all members of 'likely good' and simulated bad clusters. A logistic model was fitted modelling bad cluster status relative to good cluster status for (top) clusters without females, or (bottom) clusters including at least one record identified as being from a female. Here, logistic scores are plotted for each of the three groups. The dashed vertical line is at -1.5 in both models, a position chosen empirically as suitable for discrimination of good from bad clusters.

Table 3 shows model parameters from the derived classifier (similar estimates were obtained from a large number of other random samples). Table 5 shows the performance of the classification model when empirically regarding model scores of >-1.5 as bad (Figure 3), in an independent sample of 25,000 additional clusters (Table 6). In this validation set, over 97% of the simulated 'bad' clusters were detected by the classifier. About 5% of the 'good' clusters were classified bad, but this probably overestimates the false positive rate of the classifier, since specific identification of 'good' clusters by rules proved difficult; visual inspection of the misclassified 'good' clusters indicates that >80% probably do not represent one single individual, i.e. they are not really 'good'. Thus, we estimate the classification system detects over 97% of bad clusters, with an approximate 1% false positive rate.

**Table 5:** Multivariate Logistic model classifying bad clusters from good

			Model: Any Females		Model: No females	
Parameter	Example inputs	Distance function	Coefficient	*p*	Parameter	Example inputs
***constant***			-3.33	<1 × 10^-16^	***constant***	
***Date of birth, day***	02, 24	Levensthein	0.25	5 × 10^-4^	***Date of birth, day***	02, 24
***Date of birth, month***	01, 11	Levensthein	0.43	1 × 10^-4^	***Date of birth, month***	01, 11
***Date of birth, year***	1969, 2007	Levensthein	5.12	<1 × 10^-16^	***Date of birth, year***	1969, 2007
***Forename***	John, Chris	Jaro-Winkler	2.45	<1 × 10^-16^	***Forename***	John, Chris
***Hospital Number***	110111 or 223456	Jaro-Winkler	-0.56	<1 × 10^-16^	***Hospital Number***	110111 or 223456
***Surname***	Smith, Jones	Jaro-Winkler	not present	-	***Surname***	Smith, Jones
***Sex***	M, F	Levensthein	0.80	<1 × 10^-16^	***Sex***	M, F

A random sample of 25,000 clusters was obtained after initial record linkage. These clusters were divided into those which, on the basis of a series of rules, were thought to represent one individual ('good'), or the others ('uncertain'). The uncertain records were not used in model generation. Good clusters were then combined randomly creating a new set of clusters ('bad'). Maximal distances were computed by pairwise comparison of good and bad clusters, and a logistic model was fitted modelling bad cluster status relative to good cluster status for clusters without females, or for clusters including at least one record identified as being from a female, with backwards selection based on AIC. In the female model, surname was omitted; in the non-female model, there is only one level for the Sex field, which was therefore omitted. A model fitted is shown; very similar estimates were obtained from a large number of other builds with different random samples. *p *refers to the null hypothesis that the coefficient is zero.

**Table 6:** Classifier performance on an independent validation set of 25,000 complex clusters

Status	Predicted not bad	Predicted bad	Total	% predicted bad
Unknown	5623	2501	8124	30.7
Good	15975	901	16876	5.33
Bad	337	11698	12035	97.2

The logistic classifier derived to identify bad clusters (not bad refers to a single individual within a cluster, bad refers to more than one individual), shown in Table 4, was applied to a further random sample of 25,000 clusters obtained after initial record linkage. These were classified into 'good' 'unknown status' and 'bad' using rules, as described in Table 4 Legend and methods. The classifier performance on this validation set is shown.

### Resolution of bad clusters

The logistic classifier suggested 44,330 (1.2%) were derived from more than one individual ("bad"). As described in Methods, we developed and tested by simulation a cluster partitioning algorithm which aimed to detect single, defective identifiers combining two clusters. In a simulation, which used real clusters combined randomly using a single identifier (see Methods), of 19,863 combined clusters, 19,258 cases were correctly partitioned. Thus, ~96.9% of bad clusters of this type can be successfully resolved by the algorithm described. We incorporated the cluster resolution algorithm into the overall pipeline (see Methods). After the cluster resolution operation, only 5,570 (0.15%) were classified bad on re-scoring by the same algorithm.

### Assessment of process overall

We wished to investigate the overall performance of the linkage algorithm. We considered three aspects: specificity, sensitivity, and speed.

#### Linkage specificity

Non-specific linkage refers to placing multiple individuals in the same cluster ('identity collision'). The logistic classifier suggested that at the end of the linkage process there may be 5,570 such clusters among the 3.6 M clusters, or 0.15%. A process of manual inspection was used to inspect a random sample of these clusters. Of 100 such clusters examined by one author (DW), 6 (6%) were thought to represent two individuals, while the rest were thought to represent one individual, with a variety of variants of identifiers, name variants etc which led to false positive classification. Examples causing false classification of a clusters as containing >1 individual included incorrect NHS numbers (which are heavily weighted in the logistic classifier), and variants of forenames and surnames (particularly where double-barrelled names are variably used, or where transliteration from the original language into latin script is needed. Thus, estimated in this way, the true number of clusters containing multiple individuals in the database may be as low as 300-400. However, the logistic classifier is estimated to be only about 97% sensitive at detecting bad clusters, about 3% of true bad clusters will be missed. The classifier was applied to the 284,636 clusters with some intra-cluster variation, so another ~8,400 bad clusters might be undetected, leaving ~9,000 remaining clusters (~0.2% of the 3.6 M clusters) with identity collision in the database.

We conducted two additional automated tests of linkage specificity. Firstly we determined the numbers of clusters with multiple, theoretically unique identifiers. Secondly, we measured the numbers of patients with multiple death dates recorded. Death date is recorded in the PAS system, so having multiple death dates reflects having two PAS entries. There are two possible explanations for multiple identifiers or death dates:

• the information systems contain details on the same individual, but with different identifiers, or

• the information systems contain details on different individuals, each with their own identifiers; however, these are incorrectly clustered together.

Although this approach lacks a gold standard - we do not know how many different variants of (say) name and date of birth are expected in a given individual - it does allow quantitation of the impact of the cluster resolution operation on the 9.2 M records (Table 7). It can be seen that, in general, the greatest drops in numbers of clusters with multiple identifiers occurred for identifiers which are recognised to change little, such as forename (in contrast to surname, which changes on marriage), For NHS number, and less in poor quality identifiers (such as hospital number, which is often not unique in our hospital). In particular, there was a large drop in the number of clusters with multiple deaths remaining after collision resolution. We inspected these clusters visually. The 107 clusters with multiple deathdates could be classified into three groups. 32 (30%) were found to contain the same person with multiple PAS entries containing typographical errors. 60 (56%) errors were due to two different people sharing a common identifier. It is notable that 80% of these were records from before 2003, when data quality and completeness of identifiers were lower. The remaining 15 (14%) errors appeared to arise from laboratory records being assigned to a wrong PAS entry with similar names, a manual process which occurred in some laboratories during specimen entry. Overall, we concluded that the cluster resolution operation produced large improvements in quality of linkage.

**Table 7:** Effect of collision resolution

	Before collision resolution	After collision resolution	% drop		Before collision resolution
Number of clusters	3557951	3618233		Number of clusters	3557951
Clusters with multiple:				Clusters with multiple:	
NHS numbers	6202	2122	~66%	NHS numbers	6202
hospital numbers	97071	94238	~3%	hospital numbers	97071
birthdates	58293	35523	~39%	birthdates	58293
deathdates	830	107	~87%	deathdates	830
genders	81118	61337	~24%	genders	81118
forenames	59426	16873	~71%	forenames	59426
surnames	189657	151593	~25%	surnames	189657

After initial linkage, a process of collision resolution is applied (see methods). This causes a decrease in the number of clusters containing multiple identifiers, as detailed above.

Finally, we considered clusters which have both a microbiology sample and a PAS record; this is a large group which includes all inpatients who have ever had a microbiology sample. We identified all those who appeared to have had microbiology samples taken >7 days before they were born (according to their PAS entry), or who had samples taken >7 days after they were reported to have died. The 7-day cut off is arbitrary, and is used to select events which are unlikely to be physiological. Of 281 cases, we found 139 (49%) where there were with no differences in identifiers within the cluster, and 142 (51%) with differences in identifiers within the cluster. Careful inspection suggests that 14/142 are caused by combining two individuals inappropriately, with the other cases being due to typographical errors in dates of collection or dates of death.

Overall, we concluded that linkage specificity is high, with ~99.8% of clusters containing, with high likelyhood, only the records of one individual; the numbers of clusters containing mislinked individuals lying between ~300 and ~9,000 out of 3.6 M. Additionally, for studies where date of death is an important outcome, it appears that mislinkage is only a minor contributor to errors in reported death date, being responsible for about 10% of a series of errors identified.

#### Linkage sensitivity

We also examined linkage sensitivity. By sensitivity, we mean that all the records of one individual are partitioned into a single cluster. To determine whether it was likely that the records from a single individual were distributed across two clusters, we used a combination of a fuzzy searching method (see Methods), and manual curation.

We investigated clusters containing at least one Patient Administration system (PAS) record, a clinically and epidemiologically important group which represents an important test of the linkage system, as it includes inpatient visits and large numbers of laboratory and other records. A random sample of 250 clusters was compared with all other clusters containing at least one PAS record. This indicated that approximately 7% of clusters containing a PAS record were similar to another cluster containing a PAS record. Notably, it appeared that where there were 'duplicate' PAS records, one was created on one single hospital visit and usually lacked an NHS number, whereas all other hospital records of the patient were assigned to the other PAS entry which contained all the other hospital admission information (not shown). Likewise, approximately 14% of clusters appeared similar to clusters with no PAS record. In many of these cases, the similar records were derived from the LIMS data source, where identifiers were few (Table 4), and after examining similarities manually, it was difficult to be sure whether the observed similarities reflected two patients with similar identifiers, or one patient with typographical errors in the identifier.

#### Speed

Finally, we assessed performance. Using a single Windows 2003 server, timings for *de novo *linkage of a 9.2 M record set, broken down by step, are shown in Table 8. As can be seen, the process of linking and cluster quality control takes about 30 minutes. Custom implementations in C would likely offer higher performance. Adding records incrementally is also possible, although we have not incorporated this into our current production system, an addition rate of ~ 100,000 records per minute can be achieved when adding new data to an existing database.

**Table 8:** Overall performance

Process	Operation	Timing
1	Identifier cleaning; forename/surname duplication screening	3 min
2	Construction of unique identifiers	1 min
3	Initial clustering using identifiers	7 min
4	Identity collision detection	10 min
5	Identity collision resolution	2 min
6	Identity collision reassessment	2 min

After initial linkage, a process of collision resolution is applied (see methods). This causes a decrease in the number of clusters containing multiple identifiers, as detailed above.

## Discussion

We describe an exact-match based, highly efficient linkage scheme suitable for large scale linkage of hospital records. A key requirement is that identifiers expected to be exact should exist, or can be constructed; if this requirement is met, clustering is very efficient and readily implemented. The problem inherent in the approach is 'identity collision' - the inappropriate combination of two individuals based on mis-entry of an identifier in one of them, and its coincidence with an identifier belonging to another patient - which we have demonstrated can be addressed by systematic investigation of identifiers within suspect clusters, followed by cluster partitioning. This process also finds suspect identifiers within datasets. The algorithm is rapid, and is capable of incremental updates. Testing on five data sources including 9.2 million records indicate that ~99.8% of clusters formed consist of records from 1 individual.

As far as we know the technique described is novel, and its computational efficiency makes it attractive for linking very large numbers of records rapidly, for "business intelligence" or epidemiological purposes. In particular, we have recently gained ethical and information governance approval for an anonymised extract of this database to be used for infection research, termed the Infection in Oxfordshire Research Database. The technique has some fundamental differences with probabilistic linkage algorithms:

(1) pairwise distances between all elements are not performed in the initial linkage, and blocking steps are not used;

(2) decisions about cluster quality is made on analysis of the whole cluster formed deterministically, not on pairwise comparison of records. Maximal weighted distances within a cluster are used to classify clusters into good and bad;

(3) subsequent cluster division relies on edge structure, which probabilistic linkage does not do.

Whilst we are confident that the vast majority of clusters contain only one patient, a more difficult issue concerns the situation when records from one patient are assigned to multiple clusters. We note that among the 2.26 M patients registered with the hospital's administration system, close matches were found in about 5% of clusters. Most of these appear to represent odd orphan records together with a main record to which almost all other data is attached, and so their epidemiological impact may be small for some applications. Put another way, it may be that about 5% of the patient administration system's entries are duplicates, although they differ in all of name and date of birth, hospital number and NHS number. In many cases, it is difficult to be sure whether these entries do reflect the same individual, and we did not add a fuzzy matching component to our routine pipeline, although for some applications this will prove helpful, with or without a manual curation step.

What is an acceptable level of linkage? All linkage methods have a mislinkage rate, and we would argue that the issue of 'acceptable' levels of mislinkage is highly application specific. For clinical use it can be argued that the most dangerous situation is that in which a result is assigned to the wrong patient. This is an event which is not commonly considered clinically; because some tests are highly likely to change management, there is a substantial risk of inappropriate change in therapy. By contrast, the risk associated with the test going 'missing' - not being linked to a patient - is often less, because it can usually be repeated, although there are obviously exceptions. For epidemiological purposes, whether modelling or reporting in a tabular form, the critical issue is bias associated by mislinkage, which is application and data specific. Our study of deaths suggests that this linkage method biases analysis of death following infection, one of our epidemiological goals, relatively little.

The approach presented has a number of limitations. Firstly, it is dependent on having samples with unique identifiers, and preferably multiple unique identifiers. As alluded to above, records without at least one shared identifier will not be linked using the approach shown. This situation arises relatively commonly with our LIMS dataset, which contains low numbers of identifiers, particularly prior to 2003, and will degrade the performance of many linkage algorithms. An additional fuzzy matching step would be required to merge these clusters, if one had sufficient confidence in the match, which we do not in our current application. Alternatively, a composite identifier with high cardinality suitable for incorporating into the exact matching system could be potentially be constructed using transformations designed to eliminate common spelling or other errors, e.g. the double metaphone algorithm[18]. Lack of a fuzzy matching step in the existing pipeline contributes to efficiency, but for some data sets and applications, addition of such a step may be important.

Provided unique identifiers exist, however, if one can detect records from different individuals containing a shared, erroneous identifier, then there is the opportunity to partition the clusters formed in order to drive up clustering quality. The logistic classifier used here is not necessarily the optimal tool to do this with, and other supervised classification systems might offer increased performance.

Indeed, one interesting aspect of the algorithm used here is the separation of the algorithms used for detection of bad clusters, which relies on a logistic classifier, from that used for bad cluster partitioning (which relies on graph-based edge editing), and which was designed for the situation in which identifier error is relatively rare. This setting allows quality scoring of the effect of removal of individual identifiers from the clusters. A simple heuristic is used to score the result, and although this has good performance, it is possible that other quality measures, based around inter-node distances [10], other forms of edge weighting [9], cluster entropy [11], or the maximal intra cluster distance (as in the logistic classifier used here) might offer increased performance in both partitioning and selecting records for partitioning. In situations where unique identifiers cannot be found, although initial clustering based on non-unique identifiers could be performed, large clusters would then result likely requiring more sophisticated algorithms to partition them efficiently. Future work developing these, and comparing this algorithms with probabilistic linkage, are planned.

## Conclusion

The technique describes appears to offer a simple, rapid, highly efficient two-step method for large scale linkage for some important record types, including those found in healthcare. Clustering performance is enhanced by a system for finding of erroneous identifiers and subsequent record partitioning.

## Competing interests

DW and JF have filed patents related to the technique described. Other authors declare no competing interests.

## Authors' contributions

DW proposed and developed the algorithm described and wrote the manuscript. TP and ASW proposed a model-based method of cluster quality assessment based on simulation. JF contributed to and developed the algorithm. All authors contributed to the paper. All authors have read and approved the final manuscript.

## Pre-publication history

The pre-publication history for this paper can be accessed here:

http://www.biomedcentral.com/1472-6947/11/7/prepub
